# Machine Learning in Health Economic Evaluations: Protocol for a Scoping Review

**DOI:** 10.2196/77494

**Published:** 2025-09-24

**Authors:** Hanan Daghash, Ashleigh Kernohan, Rosiered Brownson-Smith, Rohan Pandey, Ananya Ananthakrishnan, Cen Cong, Victoria Riccalton, Edward Meinert, Gurdeep S Sagoo

**Affiliations:** 1 Population Health Sciences Institute Faculty of Medical Sciences Newcastle University Newcastle upon Tyne United Kingdom; 2 Translational and Clinical Research Institute Newcastle University Newcastle upon Tyne United Kingdom; 3 Department of Primary Care and Public Health School of Public Health Imperial College London London United Kingdom

**Keywords:** machine learning, economic evaluations, health economics, cost-effectiveness analysis, economic modelling

## Abstract

**Background:**

In recent years, the development of machine learning (ML) applications has increased substantially, indicating the potential role of ML in transforming health care. However, the integration of ML approaches into health economic evaluations is underexplored and has several challenges.

**Objective:**

This scoping review aims to explore the applications of ML in health economic evaluations. This review will also seek to identify some potential challenges to the use of ML in health economic evaluations.

**Methods:**

This review will use PRISMA-ScR (Preferred Reporting Items for Systematic Reviews and Meta-Analyses Extension for Scoping Reviews) methods. The search will be conducted on MEDLINE (Ovid), Embase (Ovid), IEEE Xplore, and Cochrane Library databases. The eligibility criteria of the selection process will be based on the study types, data sources, methods, and outcomes (SDMO) framework approach.

**Results:**

The database search yielded 4141 records after removal of retractions and duplicates. Title and abstract screening of 3718 records has been completed, resulting in 30 reports retrieved for eligibility assessment. Data extraction and charting are currently in progress. The results will be published in peer-reviewed journals by the end of 2025.

**Conclusions:**

This review will help to build up the current understanding of how ML applications are integrated in health economics evaluations. This will also explore the potential barriers to and challenges of using ML in health economics evaluations.

**International Registered Report Identifier (IRRID):**

DERR1-10.2196/77494

## Introduction 

### Background

Machine learning (ML) is a growing area in economic evaluation of health care interventions, improving predictive accuracy and resource allocation [1,2]. ML is a subfield of artificial intelligence that comprises statistical techniques that allow algorithms to learn from data and improve performance without being explicitly programmed [3,4]. In contrast, health economic evaluation provides a structured approach for comparing the costs and consequences of health care interventions to provide information in identifying the most efficient use of resources [5,6]. ML models use extensive real-world data, such as patient demographics, clinical backgrounds, treatment responses, and health care resource usage, to evaluate numerous parameters. The rapid nature of ML evaluation also allows for efficient and accurate determination of the important parameters that are considered when comparing 2 health care interventions. These may include costs of medicines or technologies, prevalence of illnesses, or the effectiveness of different treatments [7-10]. ML has significant potential to enhance health economic evaluations [9-19]; however, further exploration is required to fully understand its methodological integration and practical implications.

Despite the growing interest in ML across health research, its application within the domain of the health economic evaluation process remains limited and underexplored. Existing literature tends to focus broadly on ML applications in health outcomes research, often emphasizing prediction, diagnosis, or economic impact rather than incorporating it in the health economic evaluation process [1,2,20]. For example, a scoping review reported that 42% of studies using ML in economic evaluations focused on clinical event prediction, 22% on treatment outcomes, 16% on health care resource utilization, and 3% on cost prediction [1].

Understanding how ML is currently used in health economic evaluation is essential to ensure methodology transparency and understanding how ML could be integrated into the economic evaluation. An example of this is the research that analyzed the cost-effectiveness of high-flow nasal cannula therapy versus continuous positive airway pressure for acutely ill children using ML in data analysis [10]. The analysis indicated that high-flow nasal cannula therapy is more cost-effective for male infant patients and patients without severe respiratory distress, as it has an incremental net monetary benefit of £5310 (US $6590)overall [10]. Another example is in the case of breast cancer screening in which an ML-based risk-stratified model might save £60.4-85.3 million (US $74.9-105.9 million) per year while improving health outcomes compared to traditional screening [19]. Furthermore, applying risk assessment models can save costs while increasing value by identifying patients who can be safely discharged from the emergency room [18]. Additionally, ML techniques have shown promise in addressing nonlinear relationships and high-dimensional datasets that challenge traditional regression-based approaches [9,14,15]. For example, combining linear regression with feature selection methods such as lasso, random forest, and extreme gradient boosting has improved predictive performance in modelling complex health care costs [9,14,15]. There is a growing recognition that ML could strengthen several dimensions of economic evaluation, including the ability to capture patient heterogeneity, perform risk-stratified analysis, optimize model inputs, and handle complex data [9-19].

To the best of our knowledge, there are no previous reviews that directly explore the use of ML in health economic evaluation. However, ML has the ability to improve the health economic evaluation process through analysis of patient heterogeneity, model parameter optimization, and risk-stratified analysis [10,12,16,18]. To address this gap, this review aims to examine how ML has been applied in economic evaluations in health care and to explore the potential barriers and opportunities of using ML in the field of health economic evaluation. Guided by this aim, the review is structured around the following research questions:

1. How have ML techniques been applied within the process of health economic evaluations?
2. Which technical components of full economic evaluations (eg, model calibration, parameter estimation, heterogeneity analysis, uncertainty quantification, and metamodeling) have incorporated ML methods?
3. What challenges and barriers have been reported in applying ML within health economic evaluations?

### Objectives

The aim of this scoping review is to identify ML applications in the health economic evaluation process. Specifically, it maps how ML techniques are integrated into the technical components of full economic evaluations, such as model calibration, parameter estimation, heterogeneity analysis, uncertainty quantification, and metamodeling, regardless of the clinical or health sector context. The output will focus on the methods adopted and the challenges and barriers faced.

## Methods

### Overview

This scoping review follows Levac’s [21] recommended update to the Arksey and O’Malley scoping review framework to ensure a systematic and rigorous approach to evidence synthesis [22]. The PRISMA-ScR (Preferred Reporting Items for Systematic Reviews and Meta-Analyses Extension for Scoping Reviews) guidelines inform the reporting of the review [23]. The final report will include a PRISMA-ScR checklist (Multimedia Appendix 1).

### Search Strategy and Study Selection

A comprehensive literature search will be conducted on MEDLINE (Ovid), Embase (Ovid), IEEE Xplore, and the Cochrane Library. We will consult with information specialists to improve the search strategy. The search will use Medical Subject Headings (MeSH) terms with Boolean operators (AND, OR). The preliminary search approach is shown in Multimedia Appendix 1.

### Eligibility Criteria

This review will adopt a study types, data sources, methods, and outcomes (SDMO) framework approach [24]. This framework is used to make sure that the studies included in the selection process are relevant. The eligibility criteria are presented in Table 1. We included peer-reviewed, full-text articles and relevant conference abstracts that applied ML techniques within full economic evaluations. All health care settings were eligible, with no restrictions on publication year. Only studies published in English were considered.

**Table 1:** Eligibility criteria.

Category	Inclusion criteria	Exclusion criteria
Types of studies	Quantitative studies and economic evaluations.	Opinion pieces, editorials, and studies without economic evaluation.
Types of data	All types of data, including EHRs^a^, claims databases, clinical trial data, administrative health care datasets, and simulated or hypothetical data.	None.
Types of methods	Studies that use supervised, unsupervised, reinforcement learning, or deep learning models for economic evaluations.	Studies using ML^b^ for disease prediction or clinical decision-making without economic evaluations.
Outcomes	Studies that evaluate health economic results such as ICERs^c^, QALYs^d^, utility scores, cost predictions, and health resources.	Studies without economic evaluation outcomes.

^a^EHR: electronic health record.

^b^ML: machine learning.

^c^ICER: incremental cost-effectiveness ratio.

^d^QALY: quality-adjusted life year.

### Screening and Article Selection

All references identified from database searching were first imported into EndNote [25] for reference management, with removal of duplicate references, and then uploaded to Rayyan [26]. Two independent reviewers (AK and HA) will screen titles and abstracts based on the eligibility criteria, working blinded and independently in different locations on separate copies of the database. Discrepancies will be resolved through discussion or consultation with a third reviewer.

Although a formal calibration exercise was not conducted, both reviewers (AK and HA) discussed the initial records to ensure a shared understanding of the inclusion and exclusion criteria. These criteria served as the guiding reference throughout the screening process. Consistency was maintained throughout the screening process via ongoing communication, with discrepancies resolved through discussion and, when necessary, adjudication by a third reviewer. Interrater agreement statistics were not calculated, but reviewer alignment was tracked throughout.

### Data Extraction and Analysis

A structured data extraction form will be developed a priori and piloted to ensure consistency and reproducibility in capturing relevant study characteristics and to understand the gap in applying ML in health economic evaluation. Data will be managed using Microsoft Excel, allowing for systematic organization, coding, and retrieval of key information.

Two independent reviewers will perform data extraction, ensuring interreviewer reliability. Any discrepancies in extracted data will be resolved through discussion or, if necessary, by consulting a third reviewer. The extracted information will include the key domains described in Table 2.

**Table 2:** Key domains for extracted information.

Domain	Description
Study characteristics	Authors, year of publication, country, study design, study objective, study setting, and data sources.
Economic evaluation	Type of economic evaluation, such as cost-effectiveness analysis and cost-utility analysis, in addition to how the economic evaluation was carried out (alongside a clinical trial or economic decision models).
ML^a^ methodology	Type of ML approach used, such as supervised learning, deep learning, or reinforcement learning algorithms.
Outcomes	All economic evaluation outcomes, including ICERs^b^, QALYs^c^, utility scores, cost predictions, and health care resource allocation.
Challenges and limitations	Identified ML implementation barriers such as computational complexity, data security, and data availability.

^a^ML: machine learning.

^b^ICER: incremental cost-effectiveness ratio.

^c^QALY: quality-adjusted life year.

### Data Synthesis

Data will be synthesized narratively using descriptive analysis. Studies will be categorized based on ML methodologies, economic evaluation type, and key outcomes. Narrative analysis will identify the themes that serve as opportunities or barriers to the application of ML in economic evaluations. In addition to narrative synthesis, we will also consider presenting findings using appropriate visual summaries, such as tables, flow charts, and descriptive figures.

The extracted data will be synthesized narratively using both descriptive and thematic approaches. Studies will first be categorized based on key characteristics, including study design, clinical area, data sources, ML methodology, type of economic evaluation, computational complexity, data security and availability concerns, and the context and stage of ML application within the economic evaluation process. Subsequently, a thematic analysis will be conducted to identify recurring methodological patterns, innovations, and implementation challenges. Themes will be developed inductively and refined through team discussion.

## Results

**Figure 1:**
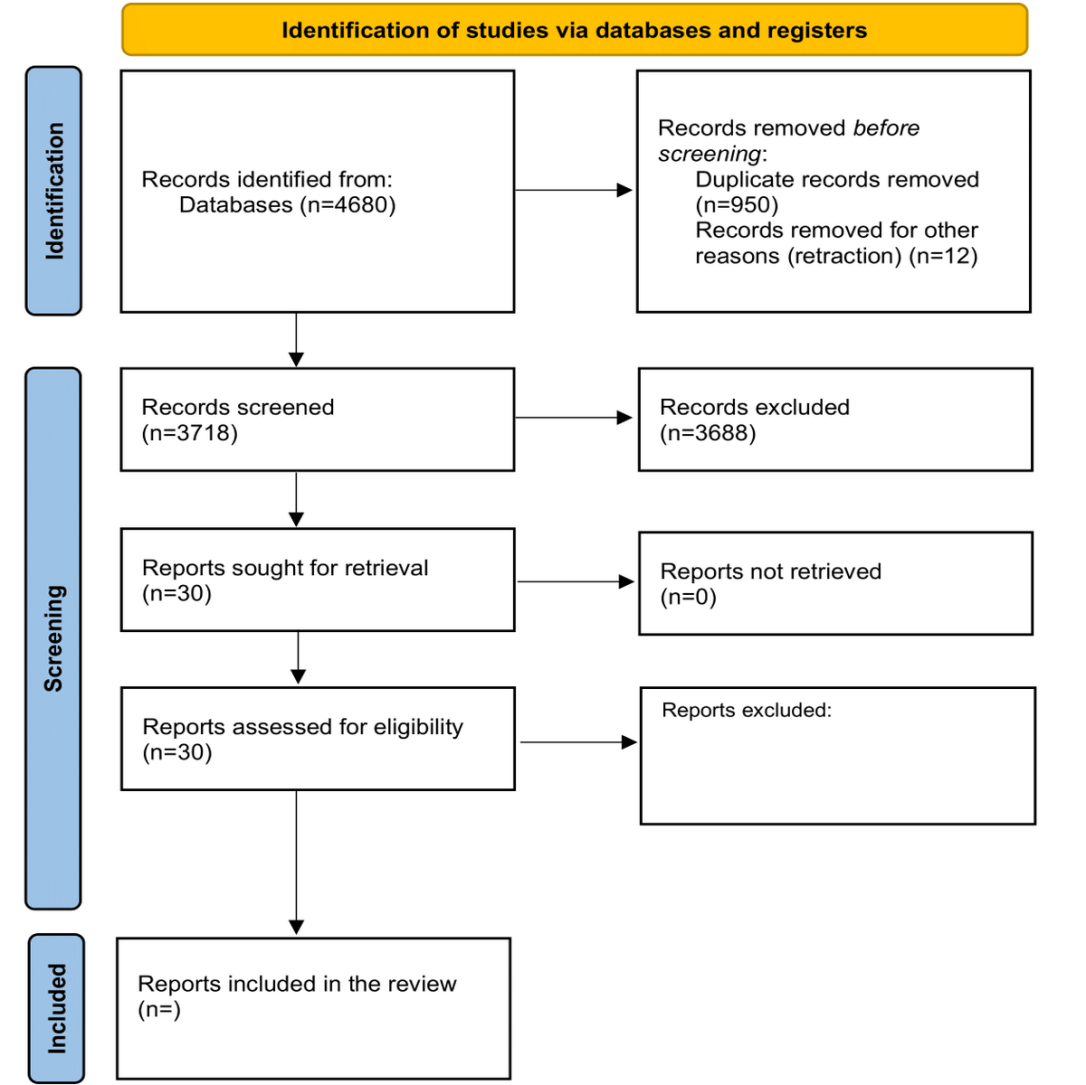
PRISMA (Preferred Reporting Items for Systematic Reviews and Meta-Analyses) flow diagram of the study selection process.

The preliminary search conducted in April 2024 yielded 4141 records from the selected databases, namely, MEDLINE (Ovid), Embase (Ovid), IEEE Xplore, and the Cochrane Library. Results will be disseminated through publication in a peer-reviewed journal by the end of 2025. The database searches are ongoing, and the PRISMA-ScR flowchart (Figure 1) illustrates the study selection process. The flowchart will be updated upon completion of full-text screening and data extraction. Based on an initial scan of titles and abstracts, the retrieved studies appeared to include a range of designs, including simulation-based modelling studies, retrospective cohorts, and hybrid designs that integrate trial data with economic models. Thematically, many studies applied ML for tasks such as model calibration, parameter estimation, heterogeneity analysis, uncertainty quantification, and metamodeling.

## Discussion

### Anticipated Findings

This scoping review is expected to identify how ML methods have been applied within health economic evaluations, particularly in areas such as model calibration, parameter estimation, heterogeneity analysis, uncertainty quantification, and metamodeling. By synthesizing this evidence, the review will map current practices and highlight methodological gaps that could inform future development of economic evaluation frameworks.

Most previous reviews examine how ML algorithms improve prediction, diagnosis, and economic impact; none have specifically explored ML in the context of the health economic evaluation process [1,2,20]. To the best of our knowledge, there is a lack of reviews on applying ML in the health economic evaluation process. This scoping review will seek to provide more comprehensive evidence on how ML is incorporated in the health economic evaluations process, what opportunities and barriers ML could face in the health economic evaluations process.

In this review, we aim to explore the existing literature in an unbiased manner; however, there are several limitations that should be acknowledged. For instance, not including grey literature may lead to missing relevant evidence from conference proceedings and other nonindexed sources. This decision was made to enhance the rigor and reliability of findings. Moreover, restricting the search to English-language publications may introduce language bias and miss relevant studies published in other languages [27]. Furthermore, this review did not assess the quality or risk of bias of the included studies, which allowed for a broad inclusion of relevant literature.

The findings of this scoping review may guide the design of future systematic reviews and comparative analyses that directly assess the effectiveness of ML-based versus traditional approaches in health economic evaluation. It may also highlight methodological areas where further research is required, such as handling of heterogeneity, validation of ML-based models, and transparency in reporting.

### Conclusions

This scoping review will explore how ML applications could be incorporated into the health economic evaluation process. This review will identify opportunities and barriers for this research. This can help us to understand the possibilities of applying ML in the health economic evaluation. By mapping current practices, the review will contribute to a clearer understanding of the role of ML in areas such as model calibration, parameter estimation, heterogeneity analysis, and uncertainty quantification. The findings will help inform the design of future systematic reviews and comparative studies, and guide methodological research aimed at strengthening the integration of ML into economic evaluation frameworks. Results will be disseminated through a peer-reviewed journal article, academic conference presentations, and professional networks in health economics and data science, and will be communicated to relevant stakeholders, including policymakers and researchers interested in ML applications in health economics.

## Appendix

Multimedia Appendix 1 PRISMA-ScR checklist and detailed search strategy.

## Abbreviations

MESH: Medical Subject Headings
ML: machine learning
PRISMA-ScR: Preferred Reporting Items for Systematic Reviews and Meta-Analyses Extension for Scoping Reviews
SDMO: study types, data sources, methods, and outcomes
