# Supplementary material for: Machine Learning in Health Economic Evaluations: Protocol for a Scoping Review
Source: JMIR Res Protoc. 2025 Sep 24;14:e77494. doi: 10.2196/77494 (PMC12508662; doi:10.2196/77494)
Supplement: Multimedia Appendix 1 [file resprot_v14i1e77494_app1.docx]

**APPENDIX A**

**Preferred Reporting Items for Systematic reviews and Meta-Analyses extension for Scoping Reviews (PRISMA-ScR) Checklist**

In the absence of a PRISMA checklist for scoping review protocols, here we adapt the PRISMA-ScR Checklist to identify the components addressed by the protocol. The final review reporting will address each item in the checklist.

| **SECTION** | **ITEM** | **PRISMA-ScR CHECKLIST ITEM** | **REPORTED** |
| --- | --- | --- | --- |
| **TITLE** | | | |
| Title | 1 | Identify the report as a scoping review. | 1 |
| **ABSTRACT** | | | |
| Structured summary | 2 | Provide a structured summary that includes (as applicable): background, objectives, eligibility criteria, sources of evidence, charting methods, results, and conclusions that relate to the review questions and objectives. | 1 |
| **INTRODUCTION** | | | |
| Rationale | 3 | Describe the rationale for the review in the context of what is already known. Explain why the review questions/objectives lend themselves to a scoping review approach. | Pg 2-3 |
| Objectives | 4 | Provide an explicit statement of the questions and objectives being addressed with reference to their key elements (e.g., population or participants, concepts, and context) or other relevant key elements used to conceptualize the review questions and/or objectives. | Aim and research questions pg.3 |
| **METHODS** | | | |
| Protocol and registration | 5 | Indicate whether a review protocol exists; state if and where it can be accessed (e.g., a Web address); and if available, provide registration information, including the registration number. | Yes  1 |
| Eligibility criteria | 6 | Specify characteristics of the sources of evidence used as eligibility criteria (e.g., years considered, language, and publication status), and provide a rationale. | Methods: Eligibility criteria pg.4 |
| Information sources* | 7 | Describe all information sources in the search (e.g., databases with dates of coverage and contact with authors to identify additional sources), as well as the date the most recent search was executed. | Methods: Search strategy and study selection pg.3 |
| Search | 8 | Present the full electronic search strategy for at least 1 database, including any limits used, such that it could be repeated. | Appendix B |
| Selection of sources of evidence† | 9 | State the process for selecting sources of evidence (i.e., screening and eligibility) included in the scoping review. | Methods: Search strategy and study selection pg.3-4 |
| Data charting process‡ | 10 | Describe the methods of charting data from the included sources of evidence (e.g., calibrated forms or forms that have been tested by the team before their use, and whether data charting was done independently or in duplicate) and any processes for obtaining and confirming data from investigators. | Methods: Data extraction pg.5 |
| Data items | 11 | List and define all variables for which data were sought and any assumptions and simplifications made. | Methods: Data extraction pg.5 |
| Critical appraisal of individual sources of evidence§ | 12 | If done, provide a rationale for conducting a critical appraisal of included sources of evidence; describe the methods used and how this information was used in any data synthesis (if appropriate). | N/A |
| Synthesis of results | 13 | Describe the methods of handling and summarizing the data that were charted. | Methods: Data synthesis pg.5-6 |
| **RESULTS** | | | |
| Selection of sources of evidence | 14 | Give numbers of sources of evidence screened, assessed for eligibility, and included in the review, with reasons for exclusions at each stage, ideally using a flow diagram. | Pg. 6 |
| Characteristics of sources of evidence | 15 | For each source of evidence, present characteristics for which data were charted and provide the citations. | N/A |
| Critical appraisal within sources of evidence | 16 | If done, present data on critical appraisal of included sources of evidence (see item 12). | N/A |
| Results of individual sources of evidence | 17 | For each included source of evidence, present the relevant data that were charted that relate to the review questions and objectives. | N/A |
| Synthesis of results | 18 | Summarize and/or present the charting results as they relate to the review questions and objectives. | N/A |
| **DISCUSSION** | | | |
| Summary of evidence | 19 | Summarize the main results (including an overview of concepts, themes, and types of evidence available), link to the review questions and objectives, and consider the relevance to key groups. | Pg 6-7 |
| Limitations | 20 | Discuss the limitations of the scoping review process. | Limitations pg.7 |
| Conclusions | 21 | Provide a general interpretation of the results with respect to the review questions and objectives, as well as potential implications and/or next steps. | Conclusion pg.7 |
| **FUNDING** | | | |
| Funding | 22 | Describe sources of funding for the included sources of evidence, as well as sources of funding for the scoping review. Describe the role of the funders of the scoping review. | Funding pg.8 |

**Appendix B**

**Detailed Search Strategy**

**Database:**
**Database:**
Ovid MEDLINE(R) In-Process & In-Data-Review Citations <1946 to April 24, 2025>
Ovid MEDLINE(R) <1946 to April Week 3 2025>
Embase <1974 to 2025 April 24>

| **#** | **Query** | **Results from 25 Apr 2025** |
| --- | --- | --- |
| 1 | Machine learning.ti,ab,kf. | 255,612 |
| 2 | ML-driven.ti,ab,kf. | 266 |
| 3 | Supervised Learning.ti,ab,kf. | 13,719 |
| 4 | exp Unsupervised Machine Learning/ | 5,277 |
| 5 | Unsupervised Learning.ti,ab,kf. | 5,968 |
| 6 | "Optimization Algorithm*".ti,ab,kf. | 13,507 |
| 7 | "Optimisation Algorithm*".ti,ab,kf. | 670 |
| 8 | "ML-based automat*".ti,ab,kf. | 39 |
| 9 | "ML-based automated".ti,ab,kf. | 27 |
| 10 | ("classification" or "random forest*" or "support vector machine" or "gradient boosting" or "xgboost" or "catboost" or "lightgbm" or "unsupervised learning" or "clustering" or "k-means" or "hierarchical clustering" or "PCA" or "dimensionality reduction" or "manifold learning" or "t-SNE" or "UMAP" or "topic modeling" or "latent class analysis" or "LDA" or "latent dirichlet allocation" or "neural network*" or "deep learning" or "semi-supervised learning" or "self-supervised learning").ti,ab,kf. | 1,754,538 |
| 11 | 1 or 2 or 3 or 4 or 5 or 6 or 7 or 8 or 9 or 10 | 1,880,217 |
| 12 | exp models, economic/ | 21,670 |
| 13 | disability-adjusted life years/ or healthy life expectancy/ or quality-adjusted life years/ | 65,783 |
| 14 | (("Personalized Medicine" or "Patient Stratification" or "clustering algorithm*") adj5 ("cost-benefit" or "quality-adjusted life years cost comparison" or "cost comparisons" or "cost-effectiveness" or "cost-minimisation" or "cost-minimization" or "cost-utility" or "cost-consequence" or "comparative effectiveness")).ti,ab,kf. | 87 |
| 15 | Cost-Utility.mp. | 22,497 |
| 16 | *Cost-Benefit Analysis/mt | 1,743 |
| 17 | *"Costs and Cost Analysis"/mt | 571 |
| 18 | *Cost-Effectiveness Analysis/ | 43,457 |
| 19 | ((economic or pharmacoeconomic or "cost-benefit" or "cost comparison" or "cost comparisons" or "cost-effectiveness" or "cost-minimisation" or "cost-minimization" or "cost-utility" or "cost-consequence") adj (model or models or modelling or analysis or analyses or evaluation or evaluations or research or study or studies)).ti,ab,kf. | 137,799 |
| 20 | 12 or 13 or 14 or 15 or 16 or 17 or 18 or 19 | 206,801 |
| 21 | 11 and 20 | 4,141 |
